# Molecular Mechanisms That Link Oxidative Stress, Inflammation, and Fibrosis in the Liver

**DOI:** 10.3390/antiox9121279

**Published:** 2020-12-15

**Authors:** Erika Ramos-Tovar, Pablo Muriel

**Affiliations:** 1 Postgraduate Studies and Research Section, School of Higher Education in Medicine-IPN, Plan de San Luis y Díaz Mirón s/n, Casco de Santo Tomás, Mexico City 11340, Mexico; erikaramost@gmail.com; 2 Laboratory of Experimental Hepatology, Department of Pharmacology, Cinvestav-IPN, Av. Instituto Politécnico Nacional 2508, Apartado Postal 14-740, Mexico City 07000, Mexico

**Keywords:** NRF2-KEAP1, inflammation, NF-κB, NLRP3 inflammasome, extracellular matrix, hepatic stellate cells, ROS, liver damage

## Abstract

Activated hepatic stellate cells (HSCs) and myofibroblasts are the main producers of extracellular matrix (ECM) proteins that form the fibrotic tissue that leads to hepatic fibrosis. Reactive oxygen species (ROS) can directly activate HSCs or induce inflammation or programmed cell death, especially pyroptosis, in hepatocytes, which in turn activates HSCs and fibroblasts to produce ECM proteins. Therefore, antioxidants and the nuclear factor E2-related factor-2 signaling pathway play critical roles in modulating the profibrogenic response. The master proinflammatory factors nuclear factor-κB (NF-κB) and the nucleotide-binding oligomerization domain (NOD)-like receptor protein 3 (NLRP3) inflammasome may coordinate to produce and activate profibrogenic molecules such as interleukins 1β and 18, which effectively activate HSCs, to produce large amounts of fibrotic proteins. Furthermore, the NLRP3 inflammasome activates pro-caspase 1, which is upregulated by NF-κB, to produce caspase 1, which induces pyroptosis via gasdermin and the activation of HSCs. ROS play central roles in the activation of the NF-κB and NLRP3 signaling pathways via IκB (an inhibitor of NF-κB) and thioredoxin-interacting protein, respectively, thereby linking the molecular mechanisms of oxidative stress, inflammation and fibrosis. Elucidating these molecular pathways may pave the way for the development of therapeutic tools to interfere with specific targets.

## 1. Introduction

Free radicals are atoms or molecules that possess unpaired electrons and are frequently unstable and highly reactive [1]. Two types of free radicals are present in biological systems: reactive oxygen species (ROS), which are radicals derived from oxygen, and reactive nitrogen species (RNS), which are derived from nitrogen. ROS can be divided into non-radicals, including hydrogen peroxide (H_2_O_2_) and singlet oxygen (^1^O_2_), and radicals, such as superoxide anion (O_2_^●−^), hydroxyl radical (^•^OH), alkoxyl radical (RO^●^), and peroxyl radical (ROO^●^). Injury to cells can be mediated by free-radical-induced lipid peroxidation, DNA strand breaks, and oxidized proteins [2,3]. ROS and RNS exhibit important physiological effects at certain levels; therefore, living organisms can regulate ROS and RNS levels via antioxidants consumed in the diet, endogenous antioxidant production, and systems specifically designed to inactivate excess radicals, to maintain a balance between antioxidants and ROS/RNS, allowing for the normal function of the organism. An imbalance favoring the accumulation of ROS/RNS is defined as oxidative/nitrosative stress. Oxidative/nitrosative stress has been demonstrated to be involved in several pathologies, including hepatic diseases [4].

## 2. Sources of ROS

Oxidative stress is a causative factor in various types of pathologies, such as cancer, diabetes, neurological disease, and liver illness [5,6,7]. However, ROS play dual roles because, at low concentrations, ROS participate in the maturation processes of cellular structures, act as second messengers in signaling pathways, and attack pathogens [7,8]. ROS can be produced in the mitochondria, where molecular oxygen (O_2_) is reduced to O_2_^●−^ by reduced nicotinamide adenine dinucleotide (NADH) and flavin adenine dinucleotide (FADH2) in a single e^−^ reduction process by complexes I and III. In addition, monoamine oxidase, α-ketoglutarate dehydrogenase, and glycerol phosphatase dehydrogenase, which are present in mitochondria, further contribute to the generation of O_2_^●−^ [8,9,10]. However, quantitatively, the endoplasmic reticulum and peroxisomes have a greater capacity to produce ROS than mitochondria in the liver [9]. Indeed, peroxisomes, microsomes, and the endoplasmic reticulum are important for the production of various kinds of ROS [9]. O_2_^●−^ can react with other molecules, to produce new, highly reactive molecules such as ^●^OH, perhydroxyl radical (HO_2_^●^), H_2_O_2_, and ^1^O_2_ [11]. Moreover, the endoplasmic reticulum can produce ROS inside the cell [12]. In addition to ROS generation in organelles, several enzymes, including cytochrome P450 (CYP) 2E1, NADPH oxidase (NOX), cyclooxygenases, xanthine oxidase, and lipoxygenases in the plasma membrane and cytosol, produce ROS [13,14]. Interestingly, NOX directly generates H_2_O_2_ or O_2_^●−^ in Kupffer cells, hepatocytes, and HSCs [15,16].

In particular, CYP enzymes play an important role in the generation of ROS in the liver [17]. These enzymes are very important in phase I metabolism of approximately three-quarters of xenobiotic metabolism reactions in humans [18]. The metabolism of drugs and other xenobiotics by CYP enzymes in the liver generates ROS and bioactivated intermediates, thus leading to oxidative stress and contributing to hepatic diseases, including alcoholic liver disease and drug-induced liver injury and cancer [12]. For a detailed review of the generation of ROS by CYP P540 enzymes, the reader is referred to References [17,19,20].

## 3. Nitrosative Stress

RNS produce a state called nitrosative stress [8], and together with ROS, they play important roles in homeostasis. Perhaps the most investigated RNS is nitric oxide (NO), which is synthesized by nitric oxide synthases (NOSs). NO production involves the 5-e^−^ oxidative process from arginine to citrulline [1,2]. NO plays an important role in the regulation of blood pressure, as it is considered to be an endothelium-relaxing factor, and it also counterattacks tumor cells, stimulates the brain, and acts as a second messenger in several pathways [21,22]. The reaction between O_2_^●−^ and NO produces peroxynitrite (ONOO^−^), which is a more reactive radical [22]. Accumulating evidence indicates that NO plays important physiological and pathological roles in the liver [23].

## 4. Role of Free Radicals in Liver Fibrosis

### 4.1. Oxidative Stress in Hepatic Fibrosis

In 1965, Comporti and colleagues were the first to describe the presence of ROS in hepatic injury induced by carbon tetrachloride (CCl_4_) intoxication, and they found elevated levels of lipid peroxidation in treated rats; in 1967, Di Luzio and Hartman also observed lipid peroxidation in fatty liver induced by ethanol. Later, in 1972 and 1984, Slater et al. proposed that ROS play a causative role in the induction of liver damage [24]. Table 1 shows a summary of the main studies that associate oxidative stress with liver diseases.

In the 1990s, investigations showed that ethanol induced ROS formation in the pathological processes of fatty liver and fibrosis development following both acute and chronic ethanol administration in experimental animals [25,26]. Alcohol abuse by humans is associated with elevated oxidative markers [27,28,29]. Moreover, protein adducts produced by the attack of membrane lipids by ROS were detected in hepatic samples from patients with elevated alcohol consumption in the areas of focal necrosis and surrounding fibrotic areas [30]. On the other hand, the overexpression of antioxidant genes was found to protect mice from oxidative stress and liver damage induced by alcohol intoxication [49,50]. Taken together, the available evidence strongly suggests that ROS and oxidative stress are implicated as pathological agents in ethanol-induced liver fibrosis. The prevalence of nonalcoholic steatohepatitis (NASH) has increased in the last two decades [51]. The metabolism of triglycerides and the imbalances in the pro-oxidant/antioxidant ratio at both the hepatic and extrahepatic levels are now well recognized as important mechanisms that are conducive to triglyceride deposition, inflammation, and fibrosis in patients with NASH [52].

A growing body of evidence indicates that the development of fibrosis in several chronic liver diseases is dependent on several factors (the multiple-hits theory) that may act in parallel. Moreover, significant perturbations in redox homeostasis, in which free radicals play a principal role, lead to scar formation and deposition within the hepatic parenchyma [24,53,54].

**Figure 1:**
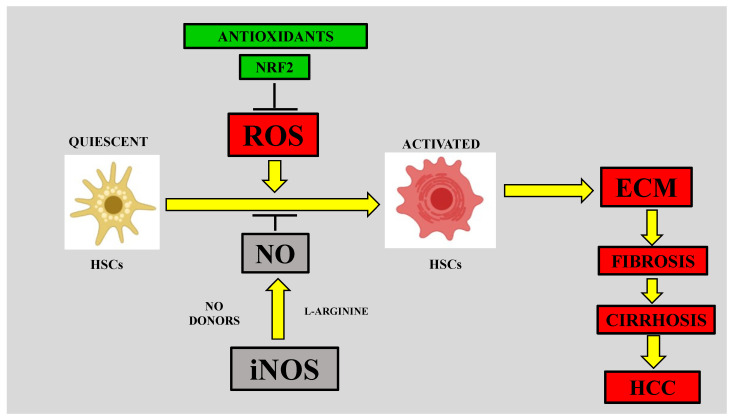
Reactive oxygen species (ROS) can induce the activation of hepatic stellate cells (HSCs), leading to the deposition of extracellular matrix (ECM) proteins, fibrosis, cirrhosis, and hepatocellular carcinoma (HCC). ROS promote the activation of HSCs from a quiescent state, which is the ECM-producing phenotype; in turn, ECM deposition leads to fibrosis, cirrhosis, and eventually HCC. Antioxidants, nuclear factor-E2-related factor-2 (NRF2) and nitric oxide (NO) seem to play antifibrotic roles by inhibiting ROS-induced HSC activation. Inducible nitric oxide synthase (iNOS) can synthesize large amounts of NO in the liver, utilizing L-arginine as a substrate.

In 1997, Cassini showed that hepatic stellate cells (HSCs) cultured with stimulated neutrophils to produce ROS showed an increase in the production of procollagen mRNA and protein, compared to HSCs co-cultured with unstimulated neutrophils; notably, this effect was abolished by incubating neutrophils with antioxidants [31]. ROS, which are produced by cytochrome P450 2E1 (CYP2E1), induce the transdifferentiation of HSCs and increase the production of collagen; again, antioxidants prevent these effects [32]. Similarly, HSCs were activated and induced to synthesize collagen I when co-cultured with Kupffer cells (KCs) in the presence of hydrogen peroxide [55]. Therefore, it seems reasonable to postulate that the induction of oxidative stress in inflammatory cells and macrophages results in the release of profibrogenic cytokines and factors. In addition to the evidence indicating the profibrotic effects of ROS in liver fibrosis, several reports have demonstrated that antioxidants or the blockade of oxidative stress induce antifibrotic effects in liver fibrosis models (Figure 1). Mice deficient in nicotinamide adenine dinucleotide phosphate (NADPH) oxidase (Nox)1 and Nox2 exhibited attenuated ROS production and hepatic fibrogenesis after CCl_4_ intoxication or bile duct ligation [33]. Knockout of the endogenous antioxidant cytoglobin (Cygb) makes mice susceptible to oxidative stress, fibrosis, and hepatocellular carcinoma induced by diethylnitrosamine (DEN) or a choline-deficient diet [56,57]. Cygb is a novel globin that has an antioxidant function in HSCs and inhibits their transdifferentiation. The ROS-scavenging function of Cygb is cytoprotective under hypoxic and oxidative stress conditions [58]. HSCs lacking the Cygb gene produce ROS and upregulate collagen, metallopeptidase inhibitor (TIMP)-1, interleukin (IL)-6, and tumor necrosis factor-alpha (TNF-α) expression.

#### Attenuating Oxidative Stress Produces an Antifibrotic Effect

Several studies have been performed to investigate the effects of various antioxidants on different models of liver damage and have shown the causative role of ROS in the profibrogenic process. In models of bile duct ligation-, CCl_4_-, and thioacetamide chronic intoxication-induced fibrosis in rodents and in vitro studies, several compounds with direct or indirect antioxidant activity have been shown to prevent or reverse the accumulation of extracellular matrix (ECM) proteins (fibrotic tissue) within the hepatic parenchyma or the production of profibrogenic genes or proteins [59,60,61,62,63,64,65,66,67,68,69,70,71,72]. One of the most studied phytodrugs with important antioxidant properties is silibinin, the main active constituent of silymarin [73]. Treatment with silibinin in human liver cirrhosis improved antioxidant status, reversed fibrosis, and stimulated regeneration [73]. Compared with placebo-treated controls, patients with cirrhosis who were treated with silibinin exhibited increases in glutathione concentrations and concurrent decreases in the N-terminal propeptide of type III collagen, a biomarker of hepatic fibrosis [74]. Moreover, decreased mortality in alcohol- and nonalcohol-induced cirrhosis patients treated with silibinin was reported [75]. Thus, evidence indicates that ROS produced in response to chronic hepatic damage induced by various causes can trigger fibrogenesis and that antioxidants, most of which originate from herbal medicines, are effective in preventing and in some cases reverting liver fibrosis [4,76].

### 4.2. Role of Nitrosative Stress in Hepatic Fibrosis

NO is synthesized in organisms by NOSs that utilize L-arginine, O_2_, and NADPH. Several isoforms of NOS have been reported, including endothelial (eNOS), inducible (iNOS), and neuronal NOS (nNOS) [77]. Moreover, eNOS and nNOS are activated by calmodulin and calcium and are constitutively expressed [78]; eNOS can also be activated by phosphorylation [79]. eNOS and nNOS produce small quantities of NO that bind to the enzyme guanylate cyclase; this enzyme produces cGMP, which regulates several signaling molecules, such as phosphodiesterases, ion channels, and protein kinases, leading to reductions in intracellular calcium levels and allowing smooth muscles to relax [80]. In contrast, iNOS is not activated by calcium and may be activated by bacterial products or cytokines in many cells, including hepatocytes, endothelial cells, macrophages, monocytes, and smooth muscle cells [78,81,82]. Although all isoforms are present in hepatic tissue, iNOS and eNOS are the most important [83]. iNOS is present in macrophages and can be induced during proinflammatory processes [84], and iNOS is also expressed in endothelial cells [85,86], hepatocytes [87,88], HSCs, and cholangiocytes [88,89]. iNOS produces much larger quantities of NO than any other isoform and lacks fine regulation; therefore, it may lead to hepatic tissue damage mainly through the nitrosylation of thiol residues. Moreover, damage due to NO is increased by oxidative stress because NO and O_2_^●−^ react to produce peroxynitrite, which is a highly reactive radical that is able to disturb several biological systems [53,90]. Nitrosative stress results from the excessive formation of RNS and perturbs several biological functions [91,92,93].

#### 4.2.1. Nitrosative Stress and HSC Activation

Activated HSCs are responsible for the production of large amounts of fibrotic tissue. Importantly, NO induces HSC contractility and proliferation, resulting in the exacerbated production of ECM proteins. Endothelins and NO act as potent modulators of HSC contractility and relaxation [94,95]. Accordingly, inhibitors of NO, Nω-nitro-L-arginine methyl ester (L-NAME), and endothelin-1 reduce the contractility of HSCs [95,96]. Activated neutrophils increase HSC collagen [97], and the administration of NO antagonists increases neutrophil accumulation in the hepatic parenchyma and exacerbates liver injury, while an increase in NO protects against damage by reducing the adhesion of neutrophils [98,99]. Moreover, it has been shown that NO and NO donors can prevent or attenuate HSC proliferation [100] (Figure 1). Platelet-derived growth factor induces the proliferation and chemotaxis of HSCs, while NO donors inhibit these effects through a pathway involving prostaglandin E2 and cAMP [101]. Interestingly, NO can prevent ROS-induced HSC proliferation [102]. A study designed to determine the role of NO in ECM deposition was performed by using a rat model of fibrosis induced by chronic CCl_4_ intoxication. In that study, the induction of NO synthesis by L-arginine partially protected the liver from the increase in collagen and reduced several markers of liver injury; in contrast, NO inhibition by L-NAME or aminoguanidine increased collagen deposition and liver damage, suggesting that endogenous NO protects the liver from injury, probably by decreasing the toxicity of free radicals and/or by attenuating the activity of HSCs [34]. Taken together, this evidence suggests that NO plays a protective role in liver damage and that the most likely mechanisms of action involve scavenging free radicals and attenuating HSC activity [53,90,102] (Figure 1).

#### 4.2.2. RNS in the Early Fibrotic Response

Most evidence indicates that NO exerts antifibrogenic effects during the early phase of fibrosis [101,103]. In the early phase of the wound-healing process, NO is produced by macrophages and KCs, contributing to the restoration of the hepatic parenchyma [104,105]. iNOS produces NO, which modulates collagen formation, cell proliferation, and wound contraction; however, the mechanism of action of NO on fibrogenesis is largely unknown [104,105]. NO downregulates type I collagen in dermal and cardiac fibroblasts [106], smooth muscle cells [107], and chondrocytes [108], and it induces antifibrotic effects in animal models of lung damage [109]. However, it has also been reported that NO promotes collagen synthesis in early traumatic wound-healing conditions [110].

#### 4.2.3. RNS in the Late Fibrotic Response

It has been noted that the beneficial effects of NO occur only at the beginning of the fibrotic process and that NO plays no role during the late phase of fibrogenesis [35]. Increased serum NO levels have been reported in cirrhotic patients, but this is considered to be the result of shear-induced eNOS activation in the hyperdynamic splanchnic circulation and not a cause of cirrhosis [111]. It is known that, in chronic liver disease, antioxidant enzymes, such as superoxide dismutase (SOD), are downregulated, which sensitizes the liver to damage induced by superoxide. In turn, hydrogen peroxide and SOD induce NO breakdown, leading to the formation of ONOO^−^. Elevated levels of serum NO have been reported in patients with cirrhosis, suggesting that NO is associated with fibrosis [111]. Patients with NASH exhibit increased levels of iNOS and nitrotyrosine, and a major role of NO during NASH-mediated fibrogenesis has been suggested [1]. iNOS deficiency in mice exerts a protective effect against CCl_4_-induced fibrosis and is mediated by a decrease in collagen synthesis in association with inhibition of ONOO^−^ formation and HSC apoptosis [36]. However, eNOS knockout in bile-duct-ligated mice indicated that fibrogenesis was not affected by this enzyme [112]. Moreover, it was reported that eNOS was not essential for the CCl_4_-induced development of fibrosis and liver damage in eNOS-knockout mice [113]. However, cirrhosis induced in rats by endotoxemia was associated with augmented S-nitrosothiol and nitrotyrosine concentrations, which are indicators of nitrosative stress [37].

#### 4.2.4. S-nitrosylation and the Liver

S-nitrosylation consists of adding a nitrosyl group to the thiol group of a cysteine to form S-nitrosothiol; this reaction is very important for the transfer of NO-mediated signals [114]. S-nitrosylation modifies the structure of proteins, which enables further posttranslational alterations, including acetylation, phosphorylation, and ubiquitination [114,115,116,117]. Notably, s-nitrosylation protects cysteine thiols against ROS attack and can scavenge NO, to prevent it from reacting with ROS, thus protecting against the formation of the deleterious and reactive radical ONOO^−^ [118]. It is known that ONOO^−^ induces the activation of MMP-2 [38], which triggers hepatic fibrosis by promoting HSC activation [119,120]. Therefore, it is reasonable to speculate that S-nitrosylation may protect the liver by reducing oxidative/nitrosative stress, preventing the activation of the proinflammatory factor nuclear factor-κB (NF-κB) and reducing scar formation by decreasing ECM-producing cells.

### 4.3. iNOS and Liver Fibrosis

iNOS is induced in liver fibrosis with diverse etiologies [121]. Compared with control mice, knockout or pharmacological inhibition of iNOS in mice leads to reduced hepatic fibrosis when the animals are challenged with cholesterol or CCl_4_, supporting the role of this enzyme and NO in the development of fibrosis [36,39]. The liver contains several kinds of cells; thus, the role of iNOS in hepatic fibrogenesis in each cell population has been investigated.

#### 4.3.1. Role of Kupffer Cell iNOS in Liver Fibrosis

Activated KCs are the major proinflammatory cytokine-producing cells in the liver; cytokines such as IL-6, TNF-α, and transforming growth factor-beta (TGF-β) activate HSCs, leading to the formation of scar tissue and liver fibrosis. Moreover, KCs can produce large amounts of NO through iNOS; in turn, NO reacts with ROS, producing RNS, such as ONOO^−^, which then can further activate HSCs, exacerbating the production of ECM. The depletion of reduced glutathione (GSH), which is responsible for reducing ROS and RNS, has been demonstrated in alcoholic liver damage [122,123,124]. The molecular mechanism by which ONOO^−^ activates HSCs needs to be further investigated.

#### 4.3.2. Role of Liver Sinusoidal Endothelial Cell (LSEC) iNOS in Liver Fibrosis

When the liver is damaged, LSECs lose fenestrae and form basement membranes, which is known as capillarization of the sinusoids [125]. This capillarization occurs earlier than fibrosis in human diseases and experimental models of hepatic chronic injury [126,127,128]. It has been reported that the capillarization of hepatic sinusoids induces the activation of HSCs and is involved in the development of liver fibrosis [129]. Interestingly, iNOS upregulation in LSECs is associated with LSEC capillarization [130,131]. It is well known that LPS and proinflammatory cytokines induce LSEC iNOS expression [85], which in turn can contribute to the capillarization of sinusoids, leading to liver fibrosis [132]. iNOS-mediated production of NO in LSECs is associated with the dysfunction of these cells [133]. However, the mechanism by which augmented iNOS expression leads to sinusoid capillarization deserves further clarification.

#### 4.3.3. Role of HSC iNOS in Liver Fibrosis

HSCs are the main ECM-producing cells and thus the major contributors to hepatic fibrosis. Quiescent HSCs become activated and transform into myofibroblast-like cells in response to profibrotic factors, leading to liver fibrosis [134,135]. The inhibition of iNOS prevents the profibrogenic process [36,39]. HSC activation and increased matrix metalloprotein (MMP)-9 expression, which play profibrogenic roles [136], occur in response to NO donors and the upregulation of iNOS in vitro and in vivo, respectively [39]. Blockade of iNOS by genetic deletion or by a specific inhibitor induced antifibrotic effects in a murine model of NASH that were accompanied by a decrease in MMP-9, suggesting an association among iNOS, MMP-9, and hepatic fibrosis [39]. The role of iNOS in fibrogenic and fibrinolytic processes deserves further investigation to clarify the role of RNS in the development of chronic liver diseases.

## 5. Nuclear Factor-E2-Related Factor-2 (NRF2) and Liver Fibrosis

### 5.1. NRF2

**Figure 2:**
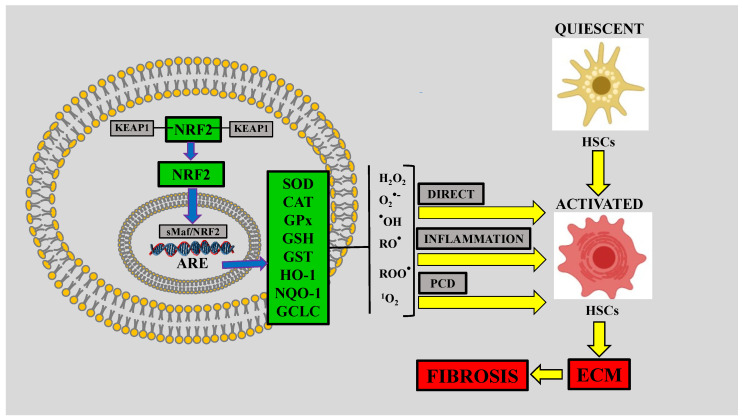
The nuclear factor-E2-related factor-2 (NRF2) pathway exerts antifibrotic effects. Reactive oxygen species (ROS), including hydrogen peroxide (H_2_O_2_), superoxide anion (O_2_^●−^), hydroxyl radical (^●^OH), alkoxyl radical (RO^●^), peroxyl radical (ROO^●^), and singlet oxygen (^1^O_2_), can activate hepatic stellate cells (HSCs) directly or by triggering inflammation or programmed cell death (PCD). In turn, activated HSCs produce large amounts of extracellular matrix (ECM) proteins that are conducive to fibrosis. Cells can regulate ROS by inducing the endogenous NRF2 antioxidant system. Inactive NRF2 is bound to the primary oxidative-stress-sensor and cytoskeleton-binding protein Kelch-like erythroid cap-n-collar (CNC) homolog-associated protein 1 (KEAP1) in the cytoplasm. Activated NRF2 is liberated from KEAP1; in turn, free NRF2 translocates to the nucleus and dimerizes with one of the small Maf (sMaf) proteins and binds to the antioxidant response element (ARE), upregulating antioxidant genes, including superoxide dismutase (SOD), catalase (CAT), glutathione peroxidase (GPx), reduced glutathione (GSH), heme oxygenase-1 (HO-1), NAD(P)H dehydrogenase (quinone 1) (NQO-1), and glutamate-cysteine ligase catalytic subunit (GCLC), which are effective in decreasing ROS; therefore, the NRF2 signaling pathway exerts an indirect antifibrotic effect.

Cells respond by expressing antioxidant and phase II detoxification enzyme genes in the presence of oxidative or nitrosative stress by interacting with the antioxidant response element (ARE) to counteract free radicals [137]. The expression of these protective genes is primarily regulated by NRF2 [138], which protects cells against ROS/RNS to maintain redox homeostasis within the cell [139]. Under physiological conditions, NRF2 is sequestered in the cytoplasm and bound to the primary oxidative-stress-sensor cytoskeleton-binding protein Kelch-like erythroid cap-n-collar (CNC) homolog-associated protein 1 (KEAP1) [140,141]. KEAP1 is a negative regulator of NRF2; however, conformational changes in the NRF2–KEAP1 complex induced by free radicals or electrophiles result in the disruption of NRF2 ubiquitination, leading to the inhibition of the proteasomal degradation pathway [142]. Upon activation, NRF2 is liberated from KEAP1; then, free NRF2 translocates to the nucleus, dimerizes with one of the small Maf proteins, and binds to AREs, upregulating protective genes such as sulfiredoxin-1, glutamate-cysteine ligase, and glutathione peroxidase-2 [141,143,144,145]. Various liver diseases involve oxidative/nitrosative stress, leading to necrosis and fibrosis [146]. Therefore, NRF2 seems to be an essential factor in fighting ROS/RNS in liver cells [139,147]. Indeed, accumulating evidence indicates that NRF2 exerts beneficial effects in several liver diseases via the induction of its target genes [4] (Figure 2).

### 5.2. Role of NRF2 in Alcoholic Liver Disease (ALD)

It is well documented that NRF2 plays a protective role in alcohol-induced oxidative stress and inflammation [148] (Figure 2). Mice lacking KEAP1 display constitutive activation of NRF2 and are less susceptible to hepatic injury induced by ethanol intoxication [40], while ethanol administration results in decreased mitochondrial GSH concentrations in mice lacking NRF2, compared with control mice [149]. Ethanol intoxication resulted in increased hepatocyte ROS production in NRF2-null mice, compared with control mice [149]. Ethanol consumption induces CYP2E1, which leads to ROS production and NRF2 activation, and is implicated in the induction of alcohol dehydrogenase after chronic ethanol treatment [150]. Increasing evidence indicates that compounds that preserve or enhance NRF2 protect the liver from ALD, indicating that NRF2 protects cells from oxidative damage and inflammation induced by alcohol intoxication [151,152,153,154,155,156,157,158]. Ethanol abuse induces alterations in lipid metabolism that are associated with the disruption of NRF2 [148]. NRF2-null mice that were stimulated with ethanol developed macrovascular steatosis and fulminant liver injury [41,42,43,44]. Serum triglycerides and liver free fatty acids were increased in NRF2-null mice but not in NRF2-overexpressing mice, compared with control mice that were administered ethanol [149].

In contrast, there is evidence that NRF2 accelerates the progression of ALD. Chronic alcohol consumption leads to the activation of HSCs, initiating the fibrogenesis phase of the disease. The induction of NRF2 with tert-butylhydroquinone (tBHQ) stimulates HSC activation and increases fibrogenic gene expression; tBHQ-mediated ARE-dependent gene induction, as well as HSC activation, was abolished when tBHQ was given in combination with a phosphatidylinositol 3-kinase inhibitor [43]. Moreover, a dominant-negative NRF2 mutant prevented the transdifferentiation of HSCs with a consequent antifibrotic effect [41]. Therefore, it seems that NRF2 can play opposite roles in different stages of ALD. Perhaps NRF2 is protective against ethanol-induced oxidative stress and inflammation, but it may play a profibrogenic role in the chronic stage of ALD. However, it is now well recognized that inflammation leads to fibrosis; thus, additional research is needed to clarify this apparent contradiction.

### 5.3. NRF2 is Decreased in NASH with Fibrosis

According to several reports, NRF2 is decreased in various models of NASH with fibrosis [159]. A lack of NRF2 increases liver damage in the high-fat diet [45], methionine- and choline-deficient diet [160,161,162], and high-fat and high-cholesterol diet [163] models of NASH, highlighting the important role of this factor in protecting against hepatic inflammation and fibrosis in NASH. Notably, NRF2 has been shown to protect against liver fibrosis due to its ability to deactivate HSCs [46] (Figure 2). The mechanism by which NRF2 decreases HSC activation is associated with the Smad signaling pathway [164]. This antifibrotic mechanism of NRF2 is very useful because, as previously mentioned, activated HSCs are the main ECM-producing cells and, therefore, are responsible for fibrogenesis in the liver (Figure 2). In this context, several authors have proposed that the most suitable strategy to prevent fibrosis may be to avoid HSC activation; moreover, the most logical mechanism to reverse the fibrotic stage is the elimination or deactivation of these cells. Interestingly, compounds with the ability to preserve/increase NRF2 activity have been shown to exert important antifibrotic effects on experimental models of liver fibrosis [4]. The induction of the NRF2 system might be a suitable option to treat NASH patients and attenuate the formation of scar tissue in these patients. Compounds that activate NRF2 can be classified into phenolic compounds with antioxidant activity (caffeic acid, butylated hydroxyanisole, and epigallocatechin-3-gallate), dithiolethiones (3H-1,2-dithiol-3-thione and oltipraz), isothiocyanates (sulforaphane), and triterpenoids (oleanolic acid) [159]. The administration of an imidazolide triterpenoid derivative that is an activator of NRF2 [165] prevented weight gain and fatty liver in mice fed a high-fat diet, and this compound was ineffective in NRF2-null mice [166]. Accordingly, another NRF2 activator, the triterpenoid 2-cyano-3,12-dioxooleana-1,9-dien-28-oic-acid, was effective in preventing inflammation and triglyceride accumulation in the liver in high-fat diet-fed mice [167]. In addition, a plant-derived NRF2 activator called sulforaphane prevented HSC activation, attenuated oxidative stress and fibrosis in the livers of mice fed a methionine- and choline-deficient diet [168], decreased the expression of α-smooth muscle actin (a marker of HSC activation) in an HSC cell line when challenged with TGF-β, and attenuated fibrosis in cholestatic mice [169]. Accordingly, activation of the NRF2 signaling pathway resulted in the prevention of oxidative stress, inflammation, and fibrosis in a NASH murine model [163].

## 6. Role of Oxidative Stress in Inflammasome-Mediated Fibrosis

**Figure 3:**
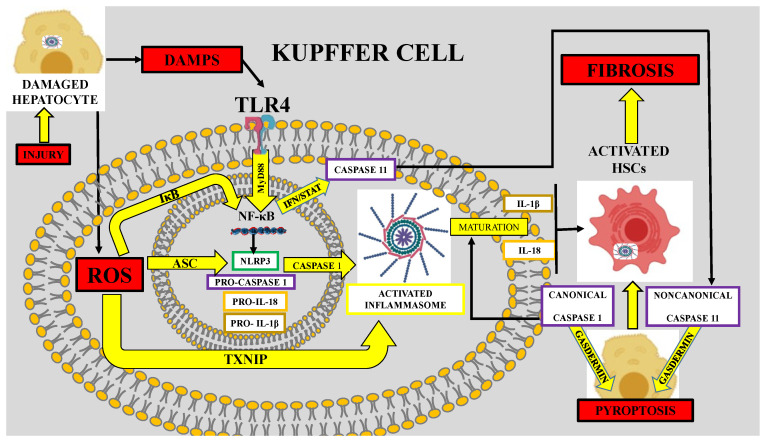
Reactive oxygen species (ROS) trigger fibrosis via several inflammatory pathways. Alcohol, viruses, or other toxic stimuli may injure hepatocytes, which release ROS and damage-associated molecular patterns (DAMPs) that bind to Toll-like receptor 4 (TLR4) in Kupffer cells. Then, the TLR4/MyD88 pathway activates nuclear factor-κB (NF-κB), which triggers the inflammatory response by promoting the transcription of the nucleotide-binding oligomerization domain (NOD)-like receptor protein 3 (NLRP3), pro-caspase-1, pro-IL-18, and pro-IL-1β. NLRP3 induces the maturation of caspase 1, which in turn triggers inflammasome activation mediated by ROS and apoptosis-associated speck-like protein containing a caspase recruitment domain (ASC); inflammasome activation induces the maturation of IL-1β and IL-18, which promote the activation of hepatic stellate cells (HSCs). ROS can activate the inflammasome through the thioredoxin-interacting protein (TXNIP) signaling pathway. NF-κB may promote caspase 11 upregulation via the IFN/STAT pathway. Caspase 1 and caspase 11 can induce pyroptosis via gasdermin by canonical or noncanonical pathways, respectively. Pyroptosis induces the activation of HSCs. ROS may promote NF-κB activation via the dissociation of the inhibitory factor IκB. Importantly, the activation of HSCs by any mechanism results in liver fibrosis.

It is now recognized that oxidative stress and inflammation are closely related to fibrotic diseases, but the mechanisms are not completely understood. In the liver, during the injury process, KCs produce large amounts of ROS in response to ethanol abuse, viruses or high-fat and -fructose diets. Oxidative stress plays an important role in the activation of the nucleotide-binding oligomerization domain (NOD)-like receptor protein 3 (NLRP3) inflammasome, the most studied cytoplasmic multiprotein complex. Damaged hepatocytes may release ROS and damage-associated molecular patterns (DAMPs). NLRP3 inflammasome signaling is initiated by ligands such as DAMPs that activate tumor necrosis factor receptor (TNFR), Toll-like receptors (TLRs), and IL receptor 1, while ROS play a fundamental role in NLRP3 inflammasome assembly and activation [150,151,152]. Myeloid differentiation factor (MyD) 88 mediates the activation of NF-κB by binding to TLR4, which induces the translocation of NF-κB to the nucleus and the transcription of pro-IL-1β, pro-IL-18, NLRP3, and interferon genes. Then, ROS produced by Nox induce the assembly of NLRP3 with the adaptor protein apoptosis-associated speck-like protein containing CARD (ASC) and recruit pro-caspase 1 [170,171,172]. Activation of the NLRP3 inflammasome via the NF-κB pathway and ROS production in KCs leads to activation of caspase-1, which mediates the maturation and secretion of IL-1β, contributing to exacerbated inflammation [171,172]. Upregulation of IL-16 and IL-17 is mediated by the NF-κB pathway, and maturation of IL-1β via the NLRP3 inflammasome triggers HSC activation with the consequent deposition of an increased amount of ECM, producing liver fibrosis. The two main pathways to fight oxidative stress are the thioredoxin system and the glutathione system. ROS stimulate thioredoxin-interacting protein, the negative regulator of the antioxidant thioredoxin, which facilitates efficient NF-κB transcription and binding with NLRP3, leading to conformational changes and activation of the NLRP3 inflammasome [48,173]. Components of the NLRP3 inflammasome and ASC are present in HSCs and play potential roles in HSC activation and liver fibrosis development. NLRP3 causes functional changes in HSCs, upregulating TGF-β and ECM production [47]. Interferon promotes the transcription of caspase-11 through the STAT pathway, which induces inflammation in HSCs, hepatocytes, and KCs through the noncanonical pyroptosis pathway and the upregulation of fibrotic markers, thus leading to liver fibrosis [174,175] (Figure 3).

In summary, NLRP3 can trigger fibrogenesis by upregulating KEAP1, thereby decreasing the antioxidant response mediated by the NRF2 signaling pathway, which leads to increased ROS levels and augmented pyroptosis, which is associated with HSC activation and exacerbated ECM deposition and fibrosis.

## 7. Conclusions and Future Perspectives

Much evidence links oxidative stress with liver fibrosis, with the former being a causative agent of the latter. KCs, resident macrophages of the liver, play a pivotal role in proinflammatory processes that may lead to HSC activation and consequently to fibrosis. ROS may activate KCs to induce the inflammatory response, which in turn activates HSCs to produce ECM proteins. Antioxidant therapy, by direct free radical scavenging or by enhancing the endogenous antioxidant machinery, has been shown to be effective in most cases in preventing/attenuating experimental fibrosis. Indeed, decreasing free radicals within the hepatic parenchyma has been proposed as a potential suitable, safe, and inexpensive therapeutic strategy against fibrosis. Some molecular mechanisms of the antifibrotic effects of free radicals are known, but most mechanisms need to be further investigated. Table 2 shows several possible molecular targets to develop antifibrotic drugs. However, additional basic research is needed to elucidate the molecular mechanisms by which ROS/RNS participate in the activation of HSCs and in several known profibrogenic signaling pathways. Significant perturbations in redox homeostasis and inflammatory and apoptosis responses are important pathologic features of human liver diseases. The most suitable strategy to prevent hepatic fibrosis may be to avoid these deleterious effects.

In addition, controlled clinical trials will provide direct evidence of the utility of improving redox homeostasis to arrest fibrosis or even to induce fibrosis remission in patients. This strategy is particularly important because, when fibrosis is advanced and cirrhosis develops, the patient has very few options for survival (organ transplantation, for example, with well-known limitations such as tissue rejection or lack of donors). In this context, antioxidants or compounds with the ability to enhance the NRF2 system appear to be suitable and less expensive options for patients suffering from chronic liver diseases of various etiologies.

## Figures and Tables

**Table 1:** A summary of the main studies that associated oxidative stress with liver diseases.

Type of Study	Model	Observed Effects	Ref.
In vivo	Acute and chronic ethanol administration	ROS participates in the pathological processes of fatty liver and fibrosis development	[25,26]
Clinical	Alcohol abuse	Elevated oxidative markers	[27,28,29]
Ex vivo	Elevated alcohol consumption	Protein adducts	[30]
In vitro	Stimulated neutrophils	ROS increases production of procollagen mRNA	[31]
In vitro	HSC and primary hepatocytes pyrazole-treated rats	Changes in collagen protein by ROS-dependent mechanisms	[32]
In vivo	Mice deficient in NOX	Exhibited attenuated ROS production	[33]
In vivo	NO inhibition	Increased collagen deposition and liver damage	[34]
In vitro	HSC incubated with ONOO^−^	NO plays no role during the late phase of fibrogenesis	[35]
In vivo	iNOS deficiency in CCl_4_-induced fibrosis	Decreased collagen synthesis; inhibited ONOO^−^ formation and HSC apoptosis	[36]
In vivo	Endotoxemia	Nitrosative stress.	[37]
In vitro	Peroxynitrite donor	Peroxynitrite may contribute to the activation of pro-MMP-2	[38]
In vivo	Blockade of iNOS	Induced antifibrotic effects	[39]
In vivo	Lacking KEAP1	Minor susceptibility to hepatic injury	[40]
In vivo	NRF2-null mice stimulated with ethanol	Developed macrovascular steatosis and fulminant liver injury	[41,42,43,44]
In vitro	Tert-butylhydroquinone	Stimulated HSC activation and increased fibrogenic gene expression	[43]
In vivo	Deletion of NRF2	Rapid onset and progression of nutritional steatohepatitis in mice	[45]
Ex vivo and in vitro	Human fibrotic liver and transient transfections of NRF2 via plasmids	Correlation between Nrf2 in HSCs and development of hepatic fibrosis NRF2 induced lipocyte phenotype in HSCs	[46]
In vitro and in vivo	Monosodium urate crystals, CCl_4_, and TAA	Inflammasome-mediated regulation of hepatic stellate cells	[47]
In vitro and in vivo	Monosodium urate crystals, H_2_0_2_ and NLRP3 activators, and Txnip–/– mice	TXNIP deficiency impaired activation of the NLRP3 inflammasome and subsequent secretion of interleukin 1β	[48]

ROS, reactive oxygen species; HSC, hepatic stellate cell.

**Table 2:** Possible molecular targets to develop antifibrotic drugs.

Target	Model	Molecule	Effect	Ref.
ROS	NASH	Vitamin E	Protected the structural components of the cell membrane from peroxidation	[176]
ROS	ALD and NALD	Silibinin	Increased glutathione concentrations, reversed fibrosis, and stimulated regeneration	[74,75]
CYP2E1	ALD	Diallyl sulfide and phenethyl isothiocyanate	Prevented the production of lipid peroxide and the accumulation of important fatty acids and reduced the pathology score	[177,178]
CYP2E1	ALD	Chlormethiazole	Reduced the proteasome proteolytic enzyme activity induced by ethanol feeding	[179]
NO	ALD	Nostrin	Decreased enzymatic activity of endothelial nitric oxide synthase	[180]
NRF2	ALD	Ethyl pyruvate	Increased anti-inflammatory factors	[181,182]
NRF2	NAFLD and NASH	NRF2 activators	Prevented inflammation, triglyceride accumulation, and fibrosis in the liver	[159,163,165,166,167,168,169].
NLPR3	NASH	MCC950	Normalized hepatic caspase 1 and IL-1β expression, plasma IL-1β, MCP-1 and IL-6, lowered ALT/AST, and reduced the severity of liver inflammation	[183]
Caspases	NASH	Emricasan	Ameliorated liver injury and fibrosis	[184]
Caspases	NASH	GS-9450	Reduced ALT levels in NASH patients	[185]
Caspases	NASH	VX-166	Inhibited collagen gene expression and reduced hepatic accumulation of cells expressing the myofibroblast marker α-SMA	[186]

NASH, nonalcoholic steatohepatitis; ALD, alcoholic liver disease; NALD, nonalcoholic liver disease; NAFLD, nonalcoholic fatty liver disease; ALT, alanine aminotransferase; AST, Aspartate aminotransferase.
